# Exercise training improves sleep pattern and metabolic profile in elderly people in a time-dependent manner

**DOI:** 10.1186/1476-511X-10-113

**Published:** 2011-07-06

**Authors:** Fábio S Lira, Gustavo D Pimentel, Ronaldo VT Santos, Lila M Oyama, Ana R Damaso, Cláudia M Oller do Nascimento, Valter AR Viana, Rita A Boscolo, Viviane Grassmann, Marcos G Santana, Andrea M Esteves, Sergio Tufik, Marco T de Mello

**Affiliations:** 1 Departamento de Psicobiologia, Universidade Federal de São Paulo, Brasil; 2 Centro de Estudo em Psicobiologia do Exercício, São Paulo, Brasil; 3 Department of Internal Medicine, State University of Campinas, Brazil; 4 Departamento de Biociência, Universidade Federal de São Paulo, Brasil; 5 Departamento de Fisiologia, Disciplina de Fisiologia da Nutrição, Universidade Federal de São Paulo, Brasil

**Keywords:** aerobic capacity, sleep, metabolism, moderate training

## Abstract

Aging and physical inactivity are two factors that favors the development of cardiovascular disease, metabolic syndrome, obesity, diabetes, and sleep dysfunction. In contrast, the adoption a habitual of moderate exercise may present a non-pharmacological treatment alternative for sleep and metabolic disorders. We aimed to assess the effects of moderate exercise training on sleep quality and on the metabolic profile of elderly people with a sedentary lifestyle. Fourteen male sedentary, healthy, elderly volunteers performed moderate training for 60 minutes/day, 3 days/week for 24 wk at a work rate equivalent to the ventilatory aerobic threshold. The environment was kept at a temperature of 23 ± 2°C, with an air humidity 60 ± 5%. Blood and polysomnographs analysis were collected 3 times: at baseline (1 week before training began), 3 and 6 months (after 3 and 6 months of training). Training promoted increasing aerobic capacity (relative VO_2_, time and velocity to VO__2_max_; p < 0.05), and reduced serum NEFA, and insulin concentrations as well as improved HOMA index (p < 0.05), and increased adiponectin levels (p < 0.05), after 3 months of training when compared with baseline data. The sleep parameters, awake time and REM sleep latency were decreased after 6 months exercise training (p < 0.05) in relation baseline values. Our results demonstrate that the moderate exercise training protocol improves the sleep profile in older people, but the metabolism adaptation does not persist. Suggesting that this population requires training strategy modifications as to ensure consistent alterations regarding metabolism.

## Introduction

Aging is characterized by several physiological and functional changes, including decline hormones, loss of muscle mass, peak oxygen uptake (VO_2peak_), and an increase of the incidence of pathologies, as such metabolic syndrome, obesity and diabetes [1-3].

Furthermore, aging induces changes in sleep with increased nighttime awakenings and arousals and decrease in deep sleep [4]. In addition, there can be increases in stage 1 and 2 sleep, decreases in stage 3 and 4 sleep, reduction rapid eye movement (REM) sleep, augment in sleep fragmentation, decreased total sleep time and sleep efficiency and increase the incidence of sleep disturbances, such as apnea and insomnia [4-6].

Another factor that frequently accompanies aging is a sedentary lifestyle. This lack of exercise increases the risk of developing cardiovascular disease and diabetes [7-10], as well as many other diseases that are linked to metabolic dysfunction. Hague et al [11] observed that, diminishment of exercise is accompanied by effects on sleep quality. Recently, our group demonstrated that acute moderate-intensity aerobic exercise appears to reduce pre-sleep anxiety and improves sleep quality in patients with chronic primary insomnia [12].

It is possible that sleep-related problems and metabolic dysfunction related with aging are at least, in part, promoted by a sedentary lifestyle [13,14]. In addition, some studies observed in sedentary healthy populations beyond 30 years of age there is a decline in VO_2peak _of near 10% [3,15].

On the other hand, acute and chronic exercise may be an option of non-pharmacological treatment for metabolic dysfunction and sleep disorders [12,16-18], especially in older individuals [19]. These effects promoted by exercise training are intensity, volume and duration dependent [20].

However, effect of moderate exercise training on metabolic and sleep profile in elderly people have been poorly investigated. Our hypothesis is that therefore, moderate exercise training improves the metabolic profile, and consequently, may induce beneficial effects on sleep parameters.

## Methods

### Subjects

The experimental protocol was approved by the Ethics Committee (number. 1592/07) of the Federal University of São Paulo in accordance with the Declaration of Helsinki. All subjects were informed of the aims and risks of the study, and their written informed consent was obtained. Fourteen male sedentary, healthy, elderly volunteers that lived independently in São Paulo, Brazil were recruited. The physical characteristics of the volunteers are presented in Table 1. All volunteers were submitted to a complete medical examination and received permission to train with a sports doctor prior to being included in the study. The exclusion criteria were the presence of cardiovascular pathologies or other diseases, preexisting or diagnosed during the clinical evaluation, that interfered with the response to training or study results.

**Table 1:** Anthropometric and training characteristics of elderly subjects that underwent of a 6-months training aerobic

Variables	Baseline	After three months	After six months
**Age (years)**	70.32 ± 0.72	---------	---------
**Height (m)**	1.68 ± 0.01	---------	---------
**Body weight (kg)**	72.88 ± 9.08	73.52 ± 8.98	73.02 ± 9.38
**Body mass index (kg/m2)**	25.20 ± 3.55	25.59 ± 3.47	25.58 ± 3.49
**Body fat (%)**	25.77 ± 7.44	26.28 ± 5.52	25.93 ± 6.57
**Absolute VO2 (L/minutes)**	2092.23 ± 445.5	2241.50 ± 394.2	2409.27 ± 420.5
**Relative VO2 (mL/kg/min)**	28.92 ± 4.82	30.54 ± 4.65*	33.77 ± 5.87*
**Time VO2 (Minutes)**	15.48 ± 1.90	16.92 ± 1.43*	18.07 ± 1.04**
**Speed VO2 (km/hour)**	6.03 ± 0.85	6.54 ± 0.66*	7.05 ± 0.61**
**VO2 Borg (Scale)**	15.23 ± 1.59	14.64 ± 1.50	15.55 ± 2.07

*p < 0.05 vs. baseline. **p < 0.01 vs. baseline.

### Experimental design and training protocol

All volunteers performed moderate exercise training bouts between 07:00-09:00. The training consisted of running for 60 minutes/day, 3 days/week for 24 wk at a work rate equivalent to their ventilatory aerobic threshold. The environment was kept at a temperature of 23 ± 2°C, with an air humidity of 60 ± 5%. Data were collected at 3 time points: at baseline (1 week before training began) at 3 and 6 months (after 3 and 6 months of training). All data were collected after 24 hours of rest to exclude any acute effects of exercise.

### Body composition

Total body mass and fat percent were measured by whole-body plethysmography (air displacement plethysmography, BOD POD body composition system; Life Measurement Instruments, Concord, CA). Height was determined by a stadiometer. In addition, the body mass index (BMI) was calculated as total body mass divided by height squared.

### Ventilatory threshold and maximal oxygen consumption

The maximal oxygen consumption (VO__2_max_) and aerobic ventilatory threshold for each volunteer was determined using an incremental exercise test on a treadmill. The initial velocity was 2.5 km/h, and the speed was increased by 1.0 km/h each 1 minute to voluntary exhaustion. Expired gas was collected in the final of the each stage to determine aerobic threshold. Respiratory and metabolic variables were obtained by measuring gaseous respiratory exchanges with a metabolic system (COSMED PFT4, Rome, Italy). The test yielded the following variables: VO__2_max_, ventilator anaerobic threshold (VATI), HR, HR at VATI, and threshold load (W). Criteria to determine oxygen consumption at VATI followed those of Wasserman et al. [21]: 1) exponential increase in ventilation; 2) abrupt increase in respiratory quotient (R); 3) systematic increase in oxygen ventilator equivalent without a change in the PEI; CO_2 _equivalent; and 4) increase in exhaled fraction of 02 (FeOV%).

### Polysomnographic recordings

Polysomnographic recordings were performed according to Pires et al. [22]. Electrode placement was carried out according to the 10-20 system. The room used for the recordings had a large comfortable bed, acoustic isolation, and controlled temperature and light. Recordings were conducted by a trained sleep technician using the digital system (Philips-Respironics, USA). The following recordings were included: electroencephalogram (C3-A2, C4-A1, O2-A1), electrooculogram, chin and tibial electromyograms, electrocardiogram, airflow (thermal sensor), thoracic-abdominal movements, a microphone placed on the lateral neck to detect snoring, pulse oximetry, and body position. Thirty-second epochs were staged according to standard criteria and visually inspected by the sleep specialist. The following parameters were analyzed: a) total sleep time (in min), defined as the actual time spent asleep; b) sleep latency (in min), defined as the time from lights out until the onset of three consecutive epochs of stage 1 or deeper sleep; c) sleep efficiency, defined as the percentage of total recording time spent asleep; d) wake after sleep onset (in min), defined as the total time scored as wakefulness between sleep onset and final awakening; e) stages 1, 2, 3, 4, and REM sleep, as percentages of total sleep time, and f) latency to REM, defined as the time from sleep onset until the first epoch of REM sleep.

### Blood collection and biochemistry determination

Blood samples were collected (20 ml) in sterile tubes containing heparin from an antecubital vein before training and 24 h after the last exercise bout after fasting 12 h. Blood samples were centrifuged at 650 × *g *for 15 minutes. Serum samples were kept at -80°C and analyzed within one week. From each sample, the serum concentrations of glucose, total cholesterol, high density lipoprotein (HDL), triacilglycerols (TG) were assessed through commercial enzymatic kits (Labtest^®^, São Paulo, Brazil). LDL cholesterol was calculated according to Friedewald et al. [23]. Non-ester fatty acid (NEFA) was assessed by colorimetric method with commercial kit (ZenBio, 3200 Chapel Hill-Nelson Blvd., Suite 104). Plasminogen activator inhibitor-1 (PAI-1) and adiponectin were assessed by ELISAs with commercial kits (R&D Systems^®^, São Paulo, Brazil). Serum insulin was quantified using enzyme-linked immunosorbent assay (ELISA), obtained from Millipore (Corp. Bedford, MA, USA).

### Statistical analyses

The data distribution was previously checked by the Bartlett's test for equal variances, and the data are reported as means and standard deviation. The differences in the plasma parameters among situations (before, after 3 and 6 exercise training) were accessed by ANOVA one way, with repeated measures and, when applicable, Tukey Post hoc was used for multiple comparisons. The analysis was carried out using STATISTICA software version 6.0 and the significance level was set at p < 0.05.

## Results

The individual physiological and anthropometric characteristics of the volunteers before, after 3 and 6 months after exercise training are described in Table 1. In addition, Table 1 shows that 3 and 6 months of moderate aerobic training was effective in improving aerobic capacity as demonstrated by the increase in VO_2max _relative (p < 0.004), time (p < 0.0001) and speedy (p < 0.0001) to VO_2 _to values before training.

Table 2 reports the results from the polysomnography. Awake time (p < 0.040) and REM sleep latency (p < 0.05) were lower only after 6 months of training, in relation baseline values. The others parameters from the polysomnography were not different.

**Table 2:** Sleep characteristics of elderly subjects that underwent of a 6-months training aerobic


Variables	Baseline	After three months	After six months

**Total sleep time (min)**	330.86 ± 60.18	342.00 ± 52.12	355.77 ± 30.64
**Sleep efficiency (%)**	78.31 ± 10.82	78.14 ± 10.20	83.05 ± 6.24
**Awake time (min)**	79.11 ± 43.98	87.87 ± 40.54	58.77 ± 20.19*
**Sleep stage 1 (%)**	7.42 ± 6.05	7.21 ± 3.23	5.95 ± 2.61
**Sleep stage 2 (%)**	57.94 ± 9.92	57.63 ± 8.00	56.38 ± 8.79
**Sleep stage 3 and 4 (%)**	12.86 ± 6.24	14.34 ± 7.06	16.92 ± 6.92
**REM (%)**	21.76 ± 8.42	20.79 ± 4.64	20.54 ± 0.93
**REM sleep latency (min)**	92.43 ± 38.40	81.18 ± 33.43	75.14 ± 31.88*

*p < 0.05 vs. baseline.

After 3 months exercise training, we found that, NEFA (see Table 3), insulin levels, and HOMA index were reduced (p < 0.05) and adiponectin was increased (p < 0.05; see Table 4), when compared with baseline data. However, these changes were no found after 6 months exercise training.

**Table 3:** Lipids profile of elderly subjects that underwent of a 6-month training aerobic


**Variables**	**Baseline**	**After three months**	**After six months**

**NEFA (μM)**	938.503 ± 251.24	664.355 ± 192.88*	821.348 ± 228.42
**TC (mg/dL)**	195 ± 36.28	197.25 ± 22.05	196.92 ± 28.94
**HDL-c (mg/dL)**	51.07 ± 9.73	51.08 ± 7.38	49.15 ± 7.98
**LDL-c (mg/dL)**	121.43 ± 29.51	122.08 ± 18.52	125.85 ± 20.05
**TG (mg/dL)**	127.71 ± 54.19	120.75 ± 38.44	110.23 ± 38.15
**PAI-1 (ng/mL)**	31.24 ± 3.55	31.27 ± 2.95	27.62 ± 4.40

*p < 0.05 vs. baseline.

NEFA: non ester fatty acid. HDL-c: high-density lipoprotein. TC: total cholesterol. TG: Triacilglycerols. LDL-c: low-density lipoprotein. VLDL-c: very-low-density lipoprotein. PAI1: plasminogen activator inhibitor type-1.

**Table 4:** Hormones related with insulin resistance of elderly subjects that underwent of a 6-months training aerobic

Variables	Baseline	After three months	After six months
**Glucose (mmol/L)**	5.56 ± 0.64	5.46 ± 0.35	5.50 ± 0.79
**Insulin (ng/mL)**	0.794 ± 0.239	0.390 ± 0.080*	0.582 ± 0.094
**HOMA-IR (index)**	4.846 ± 0.910	2.356 ± 0.452*	3.408 ± 0.547
**Adiponectin (μg/mL)**	1.554 ± 0.511	2.237 ± 0.732*	1.777 ± 0.604

*p < 0.05 vs. baseline.

No differences in plasma concentration of glucose, total cholesterol, LDL-c, HDL-c, and PAI-1 were observed (Table 3).

## Discussion

The results of the present study indicate that, elderly subjects submitted to chronic moderate training present improved aerobic capacity parameters, insulin resistance, metabolic profile, and sleep quality. Additionally, their response was time-dependent, indicating that adjusting in the workload in the exercise protocol may be necessary as to achieve maximal beneficial effects.

It is known that aging along with a sedentary lifestyle lead to loss of lean mass, reduced aerobic capacity, hormonal changes, hyperlipidemia, and sleep disorders [2,3,9,11,24,25]. In addition, all these factors contribute for the installation of several diseases, such as diabetes, obesity, and the metabolic syndrome.

Aging and sedentary lifestyle are accompanied by lower aerobic capacity [3,25-27], and this factor can be associated with reduced function capacity, compromising the performance of household chores. The etiology of the decline is multifactorial and has been attributed to the effects of biological aging (primary aging), lifestyle habits (secondary aging), and the development of subclinical and clinically apparent disease (tertiary aging) [26,28].

Aerobic exercise has long been an important recommendation for those with many of the chronic diseases typically associated with old age [29]. In the present study, we observed that the metabolic parameters related with insulin resistance were improved after 3 months exercise training. Classically, aerobic exercise training is adopted as a weight of loss program, inducing an increase in the aerobic capacity, fat oxidation by the skeletal muscle, reducing of total cholesterol, and of VLDL, TG, NEFA, all of contribute to the improvement of serum lipid profile [30,31].

The reduced NEFA levels found after 3 months exercise training may reflect the greatest uptake of fatty acids for oxidation by the skeletal muscle, corroborating the data regarding changes of aerobic capacity. However, after 6 months of exercise training NEFA levels returned to baseline values. This result may be a consequence of the adaptation of the skeletal muscle to the exercise. It is possible that intramuscular TG pool will be increased in the cells after training, and the muscle may rely, under these circumstances, rather in intramuscular TG oxidation [31,32].

Exercise training also reduced the insulin levels and improved the HOMA index, in addition to inducing increased adiponectin levels also after 3 months of the protocol.

Insulin resistance is a common condition in older persons, and the diagnosis and treatment of type 2 diabetes present unique challenges [33]. Insulin's effects on peripheral tissues (i.e., skeletal muscle, adipose tissue) involve a complex framework of signalling pathways that result in the translocation of GLUT4 transporters to the cell surface, which are responsible for the transport of glucose across the plasma membrane into the target cell [34]. An alteration in any of the related pathways reduces insulin's effectiveness and leads to the insulin-resistance and glucose intolerance associated with advancing age [35]. Adiponectin is an adipokines secreted by the adipose tissue, and has been suggested to be an important regulator of insulin action, thereby possibly linking adiposity and insulin sensitivity [36].

According the American College of Sports Medicine and the American Diabetes Association [37], exercise training is a key element in the prevention and management of type 2 diabetes. Several studies show that exercise training decreases insulin resistance and improves glucose control and diabetes, concomitantly increasing the adiponectin levels and the protein expression of insulin receptors in the skeletal muscle [18,38-40]. These effects were observed in the present study after 3 months of exercise training, but were no longer evident after 6 months. These differences may be, at least in part, consequence of not adjusting the exercise workload within the protocol.

We found reduced awake time and REM sleep latency after the protocol. Sleep is an integral part of good health and wellbeing. Pathologic disruption of sleep and variations in sleep habits are associated with a number of adverse health and safety outcomes. Obstructive sleep apnea is a very common disease, whose population prevalence is comparable to that of other important chronic diseases such as asthma, chronic obstructive pulmonary disease, type 2 diabetes, and coronary artery disease [41,42].

Loponen et al. [43] report a synergistic effect of self reported sleep problems and the metabolic syndrome on the risk of coronary heart disease (CHD) in middle aged male participants in the Helsinki Heart Study.

Driver & Taylor [44] emphasize the importance of physical exercise as a non-pharmacological treatment for sleep disorders. The mechanisms through which exercise promotes alterations in sleep architecture remain to be clarified. Researchers speculate that many hormones and metabolites may affect sleep. In fact, several studies have suggested that moderate exercise training may partially correct sleep problems [12,16,19,44-47].

It is well know that metabolic disorder is related with obstructive sleep apnea [48-51]. We suggest that improvement found in the metabolic parameters, as induced by the protocol may be involved with improve sleep quality. Nevertheless, improvement of sleep quality has only detectable after 6 months of training.

However, the schedule of training adopted in our study induced improvements in latency of REM and awake time solely and only after 6 months of the exercise training. An explanation for these results is that the volunteers presented elevated sleep efficiency for their age (about 75%). Therefore, it is possible that the effects of training may have a great impact in the elderly people with sleep complaints.

In relation to the effects only after 6 months exercise training, we speculated that, a long-term exercise is need for induced changes in sleep parameters in this population.

In conclusion, our results suggest that moderate exercise training induces recovery the some aspects of adverse sleep and metabolic in older individuals. This improvement may be partially modulated by increase in the aerobic capacity and improvement of the metabolic profile. However, more studies are required the elucidating the mechanisms involved in the beneficial effects of exercise training upon sleep and the metabolic profile in elderly people.

## Disclosure statement

All authors have contributed to the work and agree with the presented findings. Human samples were obtained, in accordance with institutional guidelines.

## Conflict of interest

The authors declare that they have no competing interests.

## Authors' contributions

FSL, GDP, RVTS, LMO, ARD, CMON, VARV, RAB, VGM, MGS, AME, ST, MTM participated or helped carry out design of the study, sample collected, assess samples, performed the statistical analysis, and writing and discussion of paper. All authors read and approved the final manuscript.
